# Making Use of Plant uORFs to Control Transgene Translation in Response to Pathogen Attack

**DOI:** 10.34133/2022/9820540

**Published:** 2022-02-03

**Authors:** Gan Ai, Jin Liu, Xiaowei Fu, Tianli Li, Hai Zhu, Ying Zhai, Chuyan Xia, Weiye Pan, Jialu Li, Maofeng Jing, Danyu Shen, Ai Xia, Daolong Dou

**Affiliations:** ^1^College of Plant Protection, Academy for Advanced Interdisciplinary Studies, Nanjing Agricultural University, Nanjing 210095, China; ^2^Department of Plant Pathology, Washington State University, Pullman, WA 99164, USA

## Abstract

Reducing crop loss to diseases is urgently needed to meet increasing food production challenges caused by the expanding world population and the negative impact of climate change on crop productivity. Disease-resistant crops can be created by expressing endogenous or exogenous genes of interest through transgenic technology. Nevertheless, enhanced resistance by overexpressing resistance-produced genes often results in adverse developmental affects. Upstream open reading frames (uORFs) are translational control elements located in the 5${}^{\prime }$ untranslated region (UTR) of eukaryotic mRNAs and may repress the translation of downstream genes. To investigate the function of three uORFs from the 5${}^{\prime }$-UTR of *ACCELERATED CELL 11* (uORFs_ACD11_), we develop a fluorescent reporter system and find uORFs_ACD11_ function in repressing downstream gene translation. Individual or simultaneous mutations of the three uORFs_ACD11_ lead to repression of downstream translation efficiency at different levels. Importantly, uORFs_ACD11_-mediated translational inhibition is impaired upon recognition of pathogen attack of plant leaves. When coupled with the *PATHOGENESIS-RELATED GENE 1* (*PR1*) promoter, the uORFs_ACD11_ cassettes can upregulate accumulation of *Arabidopsis thaliana* LECTIN RECEPTOR KINASE-VI.2 (AtLecRK-VI.2) during pathogen attack and enhance plant resistance to *Phytophthora capsici*. These findings indicate that the uORFs_ACD11_ cassettes can be a useful toolkit that enables a high level of protein expression during pathogen attack, while for ensuring lower levels of protein expression at normal conditions.

## 1. Introduction

Food production demands increase with the expanding world population and the negative impact of global climate change [1–3]. However, crop diseases become a major challenge to modern agriculture [4], with about 15% and 3% yield reduction caused by fungal/bacterial and viral pathogens, respectively [5, 6]. Microbial infection is a more severe threat to certain crops such as potato, in which it causes nearly 30% yield loss [5, 6]. The integration of enhanced resistance into new crop varieties by conventional breeding requires selection of desirable traits over several generations [7]. In contrast, ectopic expression of resistance-conferring genes is a rapid and powerful approach for enhancing crop disease resistance [4, 8].

The genetic engineering approach relies on our expanding knowledge of plant immune mechanisms [9]. There are successful examples of specifically enhancing plant resistance to certain pathogens via ectopic expression of corresponding immunity-related genes, such as the legume-like lectin receptor kinase LecRK-I.9 which is recognizing RXLR effector protein IPI-O [10] and the bacterial Elongation Factor Thermo Unstable (EF-Tu) Receptor (EFR) [11, 12]. However, overexpressing resistance-conferring genes often lead to deleterious pleiotropic effects that antagonize normal plant growth [13–15]. For example, overexpression of *Arabidopsis thaliana* NONEXPRESSER OF PR GENES 1 (AtNPR1) enhances plant disease resistance with conditional side effects [16–18]. Thus, developing novel strategies to fine-tune transgene expression and translation is critical for balancing the trade-off between plant growth and the improved defense. A promising approach is the adoption of pathogen-inducible promoters.

Genes driven by pathogen-inducible promoters are specifically induced upon pathogen infection. Thus, pathogen-inducible promoter-controlled expression of immunity-related genes may be a rational solution to reduce unnecessary growth inhibition [19–21]. For example, the promotor of Glycine max polyphenol oxidase gene, *GmPPO12*, is a pathogen-induced promoter that could be used in transgenic engineering [22]. However, pathogen-inducible promoters often auto-activate transgenes in plants [23]. For example, transgenic tobacco plants expressing the cryptogein or popA elicitor driven by the pathogen-inducible promoter *hsr203J* show broad-spectrum resistance, but some lines display runaway cell death due to hsr203J-induced gene auto-activation [24, 25]. Thus, regulatory elements with minimal side effects should be identified and used for fine-tuning transgene products at transcriptional and/or translational levels. A group of such candidates are from the translational control elements named upstream open reading frames (uORFs).

uORFs lie in the 5${}^{\prime }$ untranslated region (UTR) of eukaryotic mRNAs. They usually repress the translation of main open reading frames (mORFs), which are located downstream of uORFs [26, 27]. In Arabidopsis, there are 10,104 annotated uORFs found in about 37% of the total mRNAs [28, 29]. There are 8,531 out of 13,297 (64%) uORF-containing mRNAs harboring two or more uORFs according to the uORFlight database [30], indicating the prevalence of uORF-regulated downstream gene expression. Recent studies confirm the important roles of uORFs in regulating plant growth and defense [31–33]. In addition, uORFs have been successfully used in engineering plant immunity. For example, transgenic rice expressing AtNPR1 driven by Arabidopsis TL1-BINDING TRANSCRIPTION FACTOR 1 (AtTBF1) uORFs (uORFs_AtTBF1_) exhibits enhanced broad-spectrum disease resistance with no apparent growth retardation [34].

ACD11 is a ceramide-1-phosphate transfer protein that negatively regulates plant immunity [35, 36]. The protein stability of ACD11 is regulated by the E3 ligase XBA35.2 [37]. We previously also showed that ACD11 can be stabilized via its physical interactions with BINDING PARTNER OF ACD11 1 (BPA1)-like (BPL) family proteins [36]. However, whether there are other mechanisms regulating *ACD11* gene products is still unknown. AtLecRK-VI.2 harbors an extracellular lectin motif [38, 39] and positively regulates Arabidopsis resistance to bacterial pathogens [40]. LecRK-VI.2 also functions as a key component of systemic acquired resistance (SAR) by recognizing the putative SAR mobile signal extracellular nicotinamide adenine dinucleotide (eNAD+) [41]. Heterologous expression of LecRK-VI.2 in *N. benthamiana* increases plant resistance to a broad range of bacteria pathogens [42].

To identify uORF’s capacity of regulating downstream gene products, we have developed a fluorescence-based method to evaluate how a uORF might regulate protein production. Three predicted uORFs from the 5${}^{\prime }$UTR of *ACD11* were evaluated using this system. Individual or simultaneous mutations of the three uORFs_ACD11_ lead to repression of downstream translation variously. This translational inhibition was further impaired by pathogen inoculation. Therefore, we combined the uORFs_ACD11_ cassettes with the *PATHOGENESIS-RELATED GENE 1* (*PR1*) promoter and demonstrated that they can fine-tune AtLecRK-VI.2-mediated resistance in transgenic *N. benthamiana* plants, indicating that the uORFs_ACD11_ cassettes can be a useful toolkit for engineering crop disease resistance with desired fitness cost.

## 2. Materials and Methods

### 2.1. Plasmid Constructs

To build fluorescence- and luminescence-based reporter system, full length of GFP coding sequence (CDS), terminator of nopaline synthase gene (tNOS), CaMV 35S promoter (35S), and Luciferase CDS were amplified and inserted sequentially into the pSuper vector which contains a MAS promoter. 5${}^{\prime }$-UTR_TBF1_, 5${}^{\prime }$-UTR_ACD11_, and AtLecRK-VI.2 with a C-terminal-fused FLAG tag were amplified from WT Arabidopsis (Col-0). Site-directed mutagenesis of uORFs_ACD11_ was performed to change ATG to CTG. To generate transgenic Arabidopsis expressing NAT-GFP, 5${}^{\prime }$UTR_ACD11_, GFP, and tNOS were inserted sequentially into pSuper. To generate transgenic *N. benthamiana*, different combinations of uORFs_ACD11_ cassettes and AtLecRK-VI.2 with C-terminal fused FLAG tag and tNOS were inserted sequentially into pSuper. Primers used in this study are listed in Table S1.

### 2.2. Plant Materials and Transgenic Plants

*N. benthamiana* plants used in this study were grown in the glasshouse at 26°C under a 16-hours light/8-hours dark photoperiod for 6 weeks. *Arabidopsis* were grown at 24°C under a 12-hours light/12-hours dark photoperiod and 60% relative humidity for one month.

To generate transgenic *Arabidopsis* plants, the indicated plasmid was introduced into *Agrobacterium* strain GV3101. *Arabidopsis* WT (Col-0) plants were transformed using the standard *Agrobacterium*-mediated floral dip protocol. Transgenic plants were screened on 1/2 MS medium containing 50 mg/l hygromycin.

To generate transgenic *N. benthamiana*, the indicated plasmid was transformed into *Agrobacterium* strain GV3101. *Agrobacterium*-mediated *N. benthamiana* transformation was performed as previously described [43]. Transgenic plants were screened on 1/2 MS medium containing 50 mg/l Kanamycin.

### 2.3. *Phytophthora capsici* Culture Conditions, Culture Filtrate Acquisition and Inoculation Assay

The *P. capsici* strain LT263 used in this study was cultured and maintained at 25°C in 10% (v/v) V8 juice medium (containing 0.1 M CaCO_3_) in the dark. To produce CF, mycelium was cultured in liquid V8 medium at 25°C for 3 days. The culture was then passed through a 22 *μ*m sterile filter unit (Merck Millipore, https://www.merckmillipore.com) to generate CF. For plant infiltration, a 1/10 CF solution was used.

For mycelium inoculation, *N. benthamiana* leaves were inoculated by 5 mm disks of 4-day growth mycelium at 24 hours postinfiltration. Lesion areas were measured and photographed under UV light at 48 hpi. For zoospore inoculation on leaves of Arabidopsis, *P. capsici* mycelium was incubated in liquid medium for 3 days and then washed 3 times with distilled water. Washed mycelium was incubated in sterilized water at 25°C in darkness for 12 hours. The cultures were cold shocked at 4°C for 20 min and incubated at 25°C for 2 hours to release zoospore. Leaves were soaked in 100 spores/*μ*l *P. capsici* zoospores for 30 minutes and then held under moist conditions for subsequent analysis by confocal microscopy.

Stock solution for trypan blue staining was produced by mixing trypan blue (0.02 g), glycerol (10 ml), phenol (10 g), lactic acid (10 ml), and sterilized water (10 ml). *N. benthamiana* leaves were soaked in trypan stock solution for 24 hours at 24°C. Leaves were then destained using ethanol for 5 days. Samples were put in ethanol for taking pictures under white light.

### 2.4. Bacterial Inoculation Assay

For bacterial inoculation, 5-week-old *N. benthamiana* or 4-week-old Arabidopsis leaves were inoculated with *Pst* DC3000 or different DC3000 strains (AvrRPT2 or *hopq^1-1^*) (10^6^ cfu/ml). Bacterial population were calculated at 3 dpi.

### 2.5. Transient Expression in *N. benthamiana*

Transient expression in *N. benthamiana* was conducted as previous reported [44]. Briefly, *Agrobacterium* strains with mentioned constructs were cultured for 48 hours, collected, washed, and then resuspended in 10 mM MgCl_2_ to an optical density (OD) at 600 nm (0.4) and infiltrated into five-week-old *N. benthamiana* leaves.

### 2.6. qRT-PCR Analysis

To perform real-time PCR, total RNA was extracted by a Total RNA Kit (Tiangen Biotech Co., Ltd., Beijing, China). The cDNAs were synthesized using the HiScript II Q RT SuperMix for qPCR (Vazyme Biotech Co., Ltd., Nanjing, China). Real-time PCR was performed by using an AceQ qPCR SYBR Green Master Mix (Vazyme Biotech Co., Ltd., Nanjing, China) on an ABI Prism Q5 system. *AtUBQ10* or *NbELF18* were used as internal references. Primers used for in real-time PCR are listed in Table S1. The qRT-PCR results were concluded from three biological replicates.

### 2.7. Western Blotting and Confocal Microscopy

Plant leaves for protein extraction were ground in liquid nitrogen. Extraction buffer (0.1% Triton X-100, 150 mM KCL, 50 mM HEPES and 1 mM EDTA, protease inhibitor cocktail; pH 7.5) and 1 mM DTT was used for protein extraction. For Western blot assays, Flag, GFP (Abmart), and LUC (Sigma) antibodies were used.

To detect GFP accumulation after different treatments, confocal images were obtained at 12 hours after treatment by a confocal microscope (Zeiss LSM980, Germany). The average GFP florescence densities were quantified per 100 pixels of 20 randomly selected cells (relative unit) using ImageJ (https://imagej.en.softonic.com/).

## 3. Results

### 3.1. A Fluorescence- and Luminescence-Based Reporter System for the Function Investigation of uORFs

**Figure 1:**
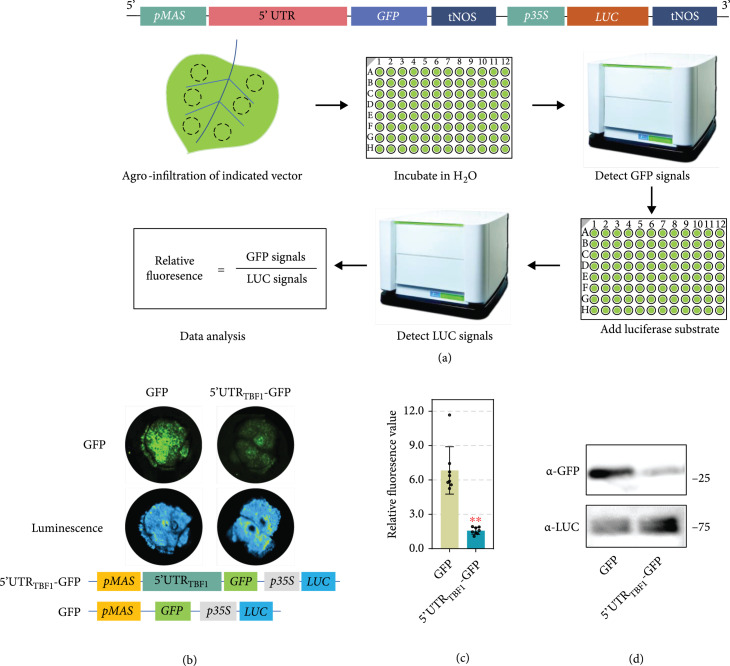
Establishment of a fluorescence- and luminescence-based reporter system to study the functions of uORFs. (a) Diagram of the fluorescence- and luminescence-based reporter system. Illustration of the constructed plasmids used in this study is shown at the top. Workflow of our system is shown below. (b) Repression of GFP production by the 5${}^{\prime }$-UTR of *TBF1*. Photos were taken 48 hours post agro-infiltration. Illustration of the indicated plasmids was shown below. (c) Relative fluorescence of leaves expressing indicated plasmids. Relative fluorescence was calculated based on the formula shown in (c) (mean±SD; n=8, Student’s t-test, P<0.01). (d) Decreased GFP protein level confirmed by Western blot assay. The GFP protein accumulation was detected by *α*-GFP antibody. Equal loading of each sample is indicated using the luciferase protein.

To investigate the function of uORFs, we designed a fluorescence- and luminescence-based reporter system to visibly measure the regulatory effect of uORFs (Figure 1(a)). In this system, the expression of *green fluorescent protein* (*GFP*) is under the control of *mannopine synthase* (*MAS*) promoter and the 5${}^{\prime }$ UTR of indicated gene. The *luciferase* (*LUC*) gene driven by CaMV *35S* promoter is used as an internal reference. GFP fluorescence and LUC luminescence intensities were quantified using a microplate reader. The relative fluorescence ratio (GFP/LUC) was calculated to remove perturbation resulted from agro-infiltration (Figure 1(a)). Notably, no significant interference was detected between green fluorescence and luminescence (Figure S1).

To check the reliability of our system, we used the previously reported *cis* translational repressor 5${}^{\prime }$-UTR_TBF1_ as a positive control [31, 34]. 5${}^{\prime }$-UTR_TBF1_ significantly repressed GFP protein accumulation in our system (Figures 1(b)–1(d)), indicating the effectiveness of this reporter system.

### 3.2. uORFs_ACD11_ Are *Cis*-Acting Elements That Repress Downstream Translation

**Figure 2:**
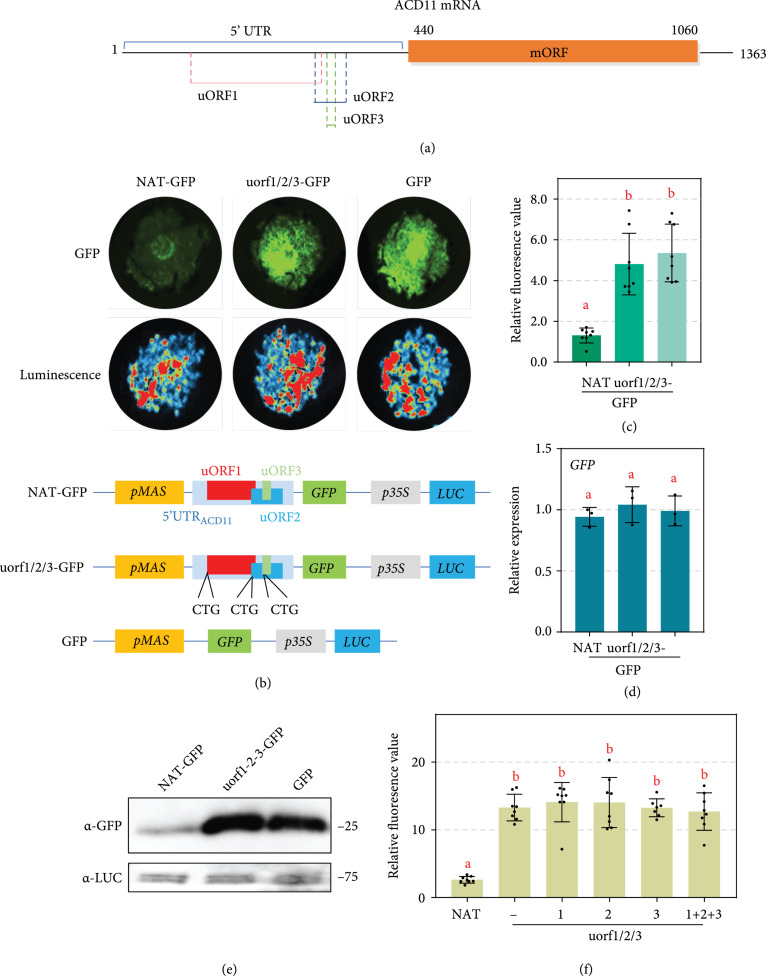
uORFs of *ACD11* are *cis*-acting elements that repress downstream translation. (a) Diagram of three uORFs identified in the 5${}^{\prime }$-UTR of *ACD11*. The main ORF (mORF) indicates *ACD11*. (b) Repression of GFP production by the 5${}^{\prime }$-UTR of *ACD11*. Photos were taken 48 hours post agro-infiltration. Illustration of the indicated plasmids was shown below. (c) Relative fluorescence of leaves expressing indicated plasmids. Relative fluorescence was calculated based on the formula shown in Figure 1(c) (mean±SD; n=8, Student’s t-test, P<0.01). (d) Relative transcript accumulation levels of *GFP*. Transcript accumulation levels of *GFP* were analyzed by qRT-PCR with *NbELF18* as internal reference. Bars represent standard errors from three biological replicates (mean±SD; n=3; ${}^{**}$, P<0.01; Student’s t-test). (e) Decreased GFP protein expression confirmed by Western blot assay. The GFP protein accumulation was detected by *α*-GFP antibody. Equal loading of each sample is indicated by the luciferase protein. (f) *ACD11* uORFs act *in cis*. uORFs was expressed *in trans* in leaves expressing uorf1/2/3 plasmid. Relative fluorescence was calculated based on the formula shown in Figure 1(c) (mean±SD; n≥8, Student’s t-test, P<0.01).

Three uORFs were identified in the 5${}^{\prime }$-UTR_ACD11_ of Arabidopsis Col-0 ecotype and were named as uORF1, uORF2, and uORF3 (Figure 2(a), Figure S2a). The Col-0 uORF1 and uORF3 were found to be conserved in 96.6% and 100% of the 1,135 accessions in the uORFlight database, respectively (Figure S3a) [30]. Two major types of uORF2 could be identified in 1,135 accessions. Except Col-0 type, Ws-2 type uORF2 contained a synonymous nucleotide substitution (G to C in the 60th base) (Figure S3a, S3b). All three uORFs_ACD11_ are highly conserved across Arabidopsis accessions which suggests that they may be functional.

A null mutant of uORFs_ACD11_ was created by mutating the start codon of all three *ACD11* uORFs to CTG (hereafter “uorf1/2/3”) and constructed into our vector. 5${}^{\prime }$-UTR_ACD11_ with native uORFs_ACD11_ (hereafter “NAT”) was used as a control (Figure 2(b)). When expressed in *N. benthamiana* leaves, uorf1/2/3 exhibited much stronger GFP fluorescence than NAT. In contrast, they generated similar intensities of luminescence (Figure 2(b)). Similarly, GFP/LUC values showed that leaves expressing the uorf1/2/3 construct displayed significantly higher GFP fluorescence than those expressing NAT (Figure 2(c)). qRT-PCR and Western blot assays demonstrated that uorf1/2/3 and NAT constructs generate similar levels of *GFP* mRNA in *N. benthamiana* leaves (Figure 2(d)), but uorf1/2/3 expression leads to higher GFP protein accumulation (Figure 2(e)). These results indicate that uORFs_ACD11_ negatively regulate downstream protein accumulation at the translation level. Notably, the absence of 5${}^{\prime }$ UTR_ACD11_ does not change GFP transcript, protein or fluorescence level as compared to uorf1/2/3 (Figures 2(b)–2(e)), indicating that the non-uORF regions in 5${}^{\prime }$-UTR_ACD11_ may not affect the expression of downstream protein.

To test whether uORFs function as *trans*- or *cis*-elements, uorf1/2/3 construct was coinfiltrated with the constructs harboring coding cassettes of uORF1, uORF2, uORF3, or all of them. The NAT construct was used as control. GFP translation cannot be blocked via coexpression peptides of any individuals or all three uORFs (Figure 2(f)), demonstrating that uORFs act *in cis*.

### 3.3. uORFs_ACD11_ Have Variable Contributions to Translational Repression

**Figure 3:**
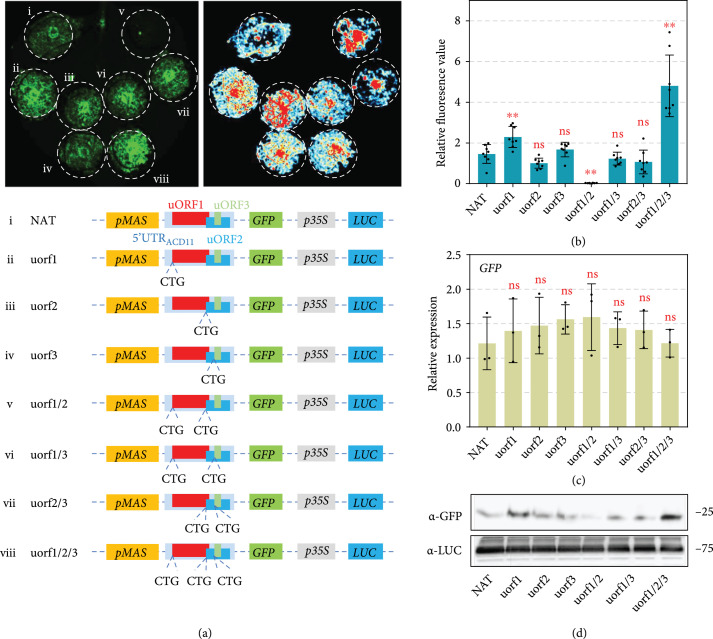
Various uORFs combinations show different downstream repressing efficiencies. (a) Repression of GFP production by *ACD11* uORF mutants. Photos were taken 48 hours post agro-infiltration. Illustration of the indicated plasmids was shown below. (b) Relative fluorescence in leaves expressing indicated plasmids. Relative fluorescence was calculated based on the formula shown in Figure 1(c) (mean±SD; n=8, Student’s t-test, P<0.01). (c) Relative transcript accumulation levels of *GFP*. Transcript accumulation levels of *GFP* were analyzed by qRT-PCR with *NbELF18* as internal reference. Bars represent standard errors from three biological replicates (mean±SD; n=3; ${}^{**}$, P<0.01; Student’s t-test). (d) Decreased GFP protein expression confirmed by Western blot assay. The GFP protein accumulation was detected by *α*-GFP antibody. Equal loading of each sample is indicated by the luciferase protein.

We next investigated the individual contributions of uORFs_ACD11_ to translational repression. A series of uORFs_ACD11_ mutants were created by individually or simultaneously mutating the start codon of uORF1, uORF2, and/or uORF3 to CTG (Figure 3(a)). These mutants exhibited comparable luminescence signals but highly variable GFP fluorescence (Figure 3(a)). Despite their relatively weak Kozak strength (Figure S2b), all three uORFs_ACD11_ are involved in the translational repression of downstream gene (Figures 3(a)–3(d)). The presence of any single uORF_ACD11_ was able to sustain the inhibition phenotype (Figures 3(a)–3(d)). Among them, uORF1 tends to be the dominant inhibitory element. Disruption of uORF1 alone could significantly increase GFP signal, which was not observed on uORF2 or uORF3 disruption (Figures 3(a)–3(d)). Nevertheless, a mutant with intact uORF3 and the disrupted uORF1 and uORF2 leads to the lowest GFP accumulation (Figures 3(a)–3(d)), indicating the shortest 9-base-pair uORF3 has the strongest repression alone. The result is consistent with the report that uORF length is not correlated with the suppression capacity [45]. Taken together, these observations indicate that uORFs_ACD11_ have complex genetic interactions, and they are not simply additive in repressing downstream protein translation.

### 3.4. uORFs_ACD11_-Mediated Translational Repression Is Attenuated upon Pathogen Infection

**Figure 4:**
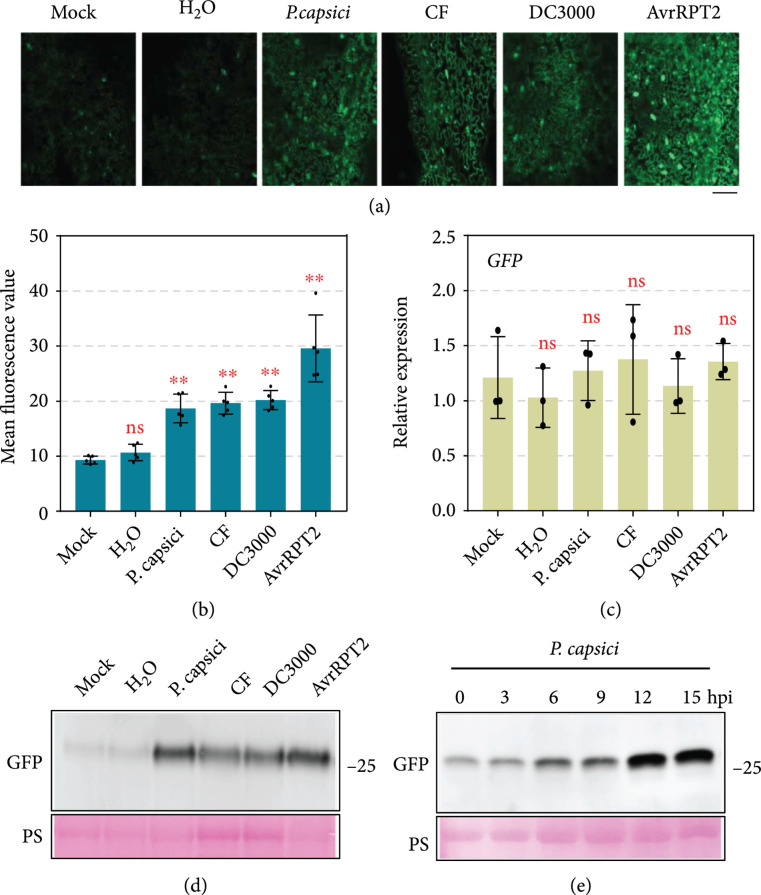
uORF_ACD11_-mediated translational repression is attenuated upon pathogen infection. (a) GFP accumulation after pathogen-related treatments. Confocal microscope images were taken 12 hours posttreatment. Bar=50 μm. (b) Mean fluorescence value of indicated leaves. The average GFP florescence densities were quantified per 100 pixels of 20 randomly selected cells (relative unit) using ImageJ. (c) Relative transcript accumulation levels of *GFP*. Transcript accumulation levels of *GFP* were analyzed by qRT-PCR with *AtUBQ10* as internal reference. Bars represent standard errors from three biological replicates (mean±SD; n=3; ${}^{**}$, P<0.01; Student’s t-test). (d) GFP protein accumulation detected by Western blot assay. The *α*-GFP antibody was used to detect the expression of GFP protein. Equal loading of each sample is indicated by Ponceau staining. (e) GFP protein accumulation changes after *P. capsici* inoculation. The GFP protein accumulation was detected by *α*-GFP antibody. Equal loading of each sample is indicated by Ponceau staining.

The 5${}^{\prime }$-UTR_ACD11_-GFP transgenic Arabidopsis plants were produced to test the response of uORFs_ACD11_ to pathogen infection (Figure S4a). GFP fluorescence generated in these transgenic plants was too weak to detect using *in vivo* fluorescence imaging (Figure S4b), but visible under confocal microscope (Figure 4(a)). Transgenic Arabidopsis leaves were challenged with the oomycete pathogen *Phytophthora capsici* (Figure S4c). GFP fluorescence was enhanced in leaves inoculated with *P. capsici* zoospores or culture filter (CF) (Figures 4(a) and 4(b)) [46]. qRT-PCR analysis showed that the transcript accumulation level of *GFP* was unchanged after inoculation (Figure 4(c)). Western blot assay confirmed that the inoculation of either *P. capsici* zoospores or CF promoted GFP accumulation at the protein level (Figure 4(d)). A time-course assay further showing that GFP protein accumulation increased over time in 5${}^{\prime }$-UTR_ACD11_-GFP transgenic Arabidopsis leaves infected by *P. capsici* zoospores (Figure 4(e)), indicating that the translational repression efficiency of uORFs_ACD11_ could be impaired upon *P. capsici*. Similar to *P. capsici*, the bacterial pathogen *Pseudomonas syringae* pv. *tomato* (*Pst*) DC3000 also induced GFP accumulation in 5${}^{\prime }$-UTR_ACD11_-GFP transgenic Arabidopsis leaves at the translation level (Figures 4(a)–4(d)). Interestingly, *Pst* DC3000_AvrRPT2_, an avirulent strain that induces hypersensitive response in Arabidopsis [47, 48], showed higher GFP-induction efficiency than the wild-type (WT) *Pst* DC3000 (Figures 4(a)–4(d)). This observation indicates that the translational repression ability of uORFs_ACD11_ can be attenuated after plant recognizing different pathogens.

### 3.5. Different uORFs_ACD11_ Cassettes Lead to Variable Accumulation of AtLecRK-VI.2 for Acquired Resistance

Since different uORFs_ACD11_ combinations showed variable downstream translational repression efficiencies, they could be used to fine-tune exogenous protein accumulation in transgenic plants, which may minimize the deleterious pleiotropic effects caused by gene overexpression [13–15] and help to balance plant growth and immunity [34].

**Figure 5:**
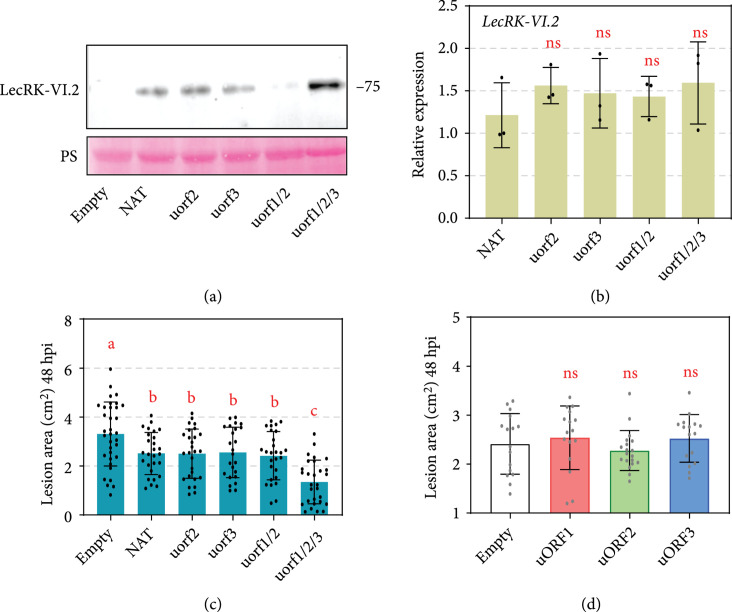
Different uORFs_ACD11_ cassettes lead to variable AtLecRK-VI.2 protein accumulation and pathogen resistance levels. (a) AtLecRK-VI.2 protein accumulation levels detected by Western blot assay. The *α*-FLAG antibody was used to detect the expression of AtLecRK-VI.2 protein. Equal loading of each sample is indicated by Ponceau staining. (b) Relative transcript accumulation levels of *AtLecRK-VI.2*. Transcript accumulation levels of *AtLecRK-VI.2* were analyzed by qRT-PCR with *NbELF18* as internal reference. Bars represent standard errors from three independent biological replicates (mean±SD; n=3; ${}^{**}$, P<0.01; Student’s t-test). (c) Expression of AtLecRK-VI.2 driven by *ACD11* uORF-mutants provides variable levels of resistance to *P. capsici* infection in *N. benthamiana*. Lesion areas were calculated at 48 hpi. The result was calculated from three independent replicates with at least five leaves per replicate (mean±SD; n≥18; ${}^{**}$, P<0.01, Student’s t-test). (d) No pathogen resistance is induced by uORF1, uORF2, or uORF3. Leaves were stained by trypan blue and destained with ethanol. Photos were taken in white light and shown in (a). Bar=2 cm. The lesion areas were calculated from three independent experiments were shown below (mean±SD; n≥18; ${}^{**}$, P<0.01, Student’s t-test).

AtLecRK-VI.2, a positive modulator of PAMP-triggered immunity (PTI) response and SAR [40–42], was selected to test its translational regulation by uORFs_ACD11_ cassettes. Constructs of AtLecRK-VI.2 under the control of uorf2, uorf3, uorf1/2/3, or NAT were transiently expressed in *N. benthamiana*. Similar to results obtained using the GFP reporter, uorf1/2 led to the lowest AtLecRK-VI.2 protein accumulation. uorf2, uorf3, and NAT resulted in moderate protein accumulation levels while the highest AtLecRK-VI.2 level was achieved by using uorf1/2/3 (Figure 5(a)). Consistent with the translational repression function of uORFs_ACD11_, no significant difference was observed on *AtLecRK-VI.2* transcription levels (Figure 5(b)).

*P. capsici* resistance levels were tested for *N. benthamiana* plants transiently expressing AtLecRK-VI.2 under the control of different uORFs_ACD11_ cassettes. Compared to empty vector, all cassettes significantly enhanced plant immunity to *P. capsici* at 48 hours postinoculation (hpi), with uorf1/2/3-LecRK-VI.2 delivering the highest resistance (Figure 5(c)). No increased resistance could be found in leaves expressing uORF1, uORF2, or uORF3 (Figure 5(d)), indicating that none of the peptides encoded by the three uORFs are directly involved in *P. capsici* resistance.

**Figure 6:**
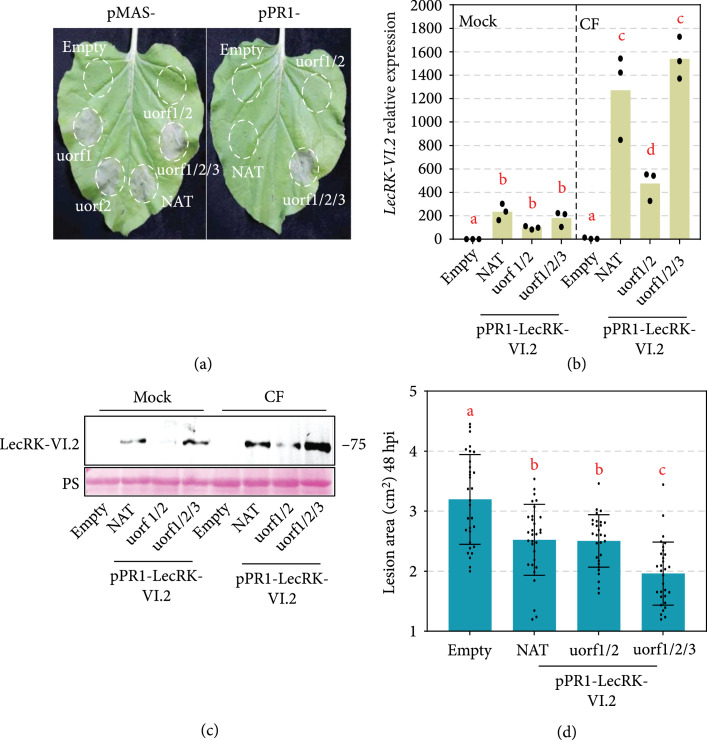
Pathogen-inducible protein accumulation of AtLecRK-VI.2 is achieved by using the combinations of different uORFs and the NbPR1 promoter. (a) Cell death induced by indicated gene products. Leaves infiltrated with Agrobacterium harboring indicated constructs. Photos were taken at 5 dpi in white light. (b) CF-induced AtLecRK-VI.2 transcript accumulation confirmed by qRT-PCR. Transcript accumulation levels of *AtLecRK-VI.2* were analyzed by qRT-PCR. *P. capsici* CF was infiltrated into leaves expressing the indicated plasmid. Total RNA was extracted 1 hour posttreatment. The *NbELF18* gene was used as an internal reference. Bars represent standard errors from three biological replicates (mean±SD; n=3; ${}^{**}$, P<0.01; Student’s t-test). (c) Induced AtLecRK-VI.2 protein accumulation confirmed by Western blot assay. CF was infiltrated into leaves expressing the indicated plasmid. Proteins was extracted 2 hours posttreatment. The *α*-FLAG antibody was used to detect the expression of AtLecRK-VI.2 protein. Equal loading of each sample is indicated by Ponceau staining. (d) Enhanced resistance to *P. capsici* infection provided by *ACD11* uORF-mutants coupled with the PR1 promoter and AtLecRK-VI.2 in *N. benthamiana*. Lesion areas were calculated from three independent experiments with at least five leaves per replicate (mean±SD; n≥18; ${}^{**}$, P<0.01, Student’s t-test).

All expression constructs except uorf1/2-LecRK-VI.2 induced intense cell death at 5 days after infiltration (dpi) (Figure 6(a)), which may be explained by the lowest LecRK-VI.2 accumulation level caused by uorf1/2.

### 3.6. Combinations of Different uORFs and the *NbPR1* Promoter Lead AtLecRK-VI.2 to Be Pathogen-Inducible

To express LecRK-VI.2 specifically in response to infection, we combined the pathogen-inducible *N. benthamiana PR1* (*NbPR1*) promoter (pPR1) with different uORFs-LecRK-VI.2 cassettes and transiently expressed them in *N. benthamiana*. *P. capsici* CF infiltration significantly enhanced LecRK-VI.2 transcript (Figure 6(b)) and protein (Figure 6(c)) accumulations in leaves expressing pPR1-NAT-LecRK-VI.2, pPR1-uorf1/2-LecRK-VI.2, or pPR1-uorf1/2/3-LecRK-VI.2 construct. Despite that pPR1-uorf1/2-LecRK-VI.2 led to the lowest LecRK-VI.2 protein accumulation, it provided similar level of *P. capsici* resistance as that of pPR1-NAT-LecRK-VI.2 (Figure 6(d)). Intense cell death was induced at 5 dpi in leaves expressing pPR1-uorf1/2/3-LecRK-VI.2 but not pPR1-uorf1/2-LecRK-VI.2 (Figure 6(a)). However, unlike NAT-LecRK-VI.2, expression of pPR1-NAT-LecRK-VI.2 did not cause necrosis in leaves, indicating that the activity of pathogen-inducible *pPR1* may confer a lower downstream gene expression level in normal conditions (Figure 6(a)).

### 3.7. pPR1-uorf1/2-AtLecRK-VI.2 Enhances *N. benthamiana* Resistance to *P. capsici* with no Apparent Suppression to Plant Growth

**Figure 7:**
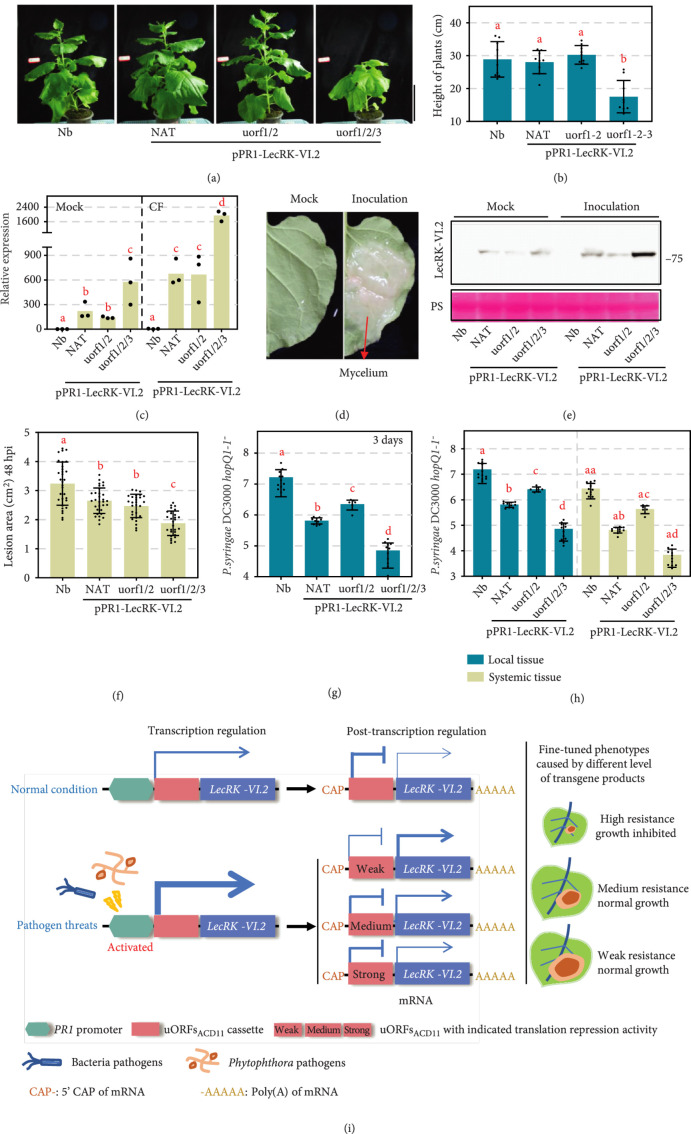
pPR1-uORFs_ACD11_ cassettes fine-tune AtLecRK-VI.2-mediated resistance to pathogens in transgenic *N. benthamiana*. (a, b) The growth phenotypes of indicated transgenic *N. benthamiana* plants. Photos were taken at 8 weeks after sowing (a). Plant heights (from soil to the top of plants) were calculated and showed in (b). (c) *AtLecRK-VI.2* transcript accumulation confirmed by qRT-PCR. Transcript accumulation levels of *AtLecRK-VI.2* were analyzed by qRT-PCR. CF was infiltrated into the indicated leaves. Total RNA was extracted 1 hour posttreatment. The *NbELF18* gene was used as an internal reference. Bars represent standard errors from three biological replicates (mean±SD; n=3; ${}^{**}$, P<0.01; Student’s t-test). (d, e) Induced AtLecRK-VI.2 protein accumulation upon *P. capsici* infection. *P. capsici* mycelium was inoculated on the indicated leaves. Proteins was extracted 12 hours postinoculation. The *α*-FLAG antibody was used to detect the expression of AtLecRK-VI.2 protein. Equal loading of each sample is indicated by Ponceau staining. (f) Enhanced resistance to *P. capsici* infection in transgenic *N. benthamiana*. Lesion areas were calculated from three independent experiments with at least five leaves per replicate (mean±SD; n≥18; ${}^{**}$, P<0.01, Student’s t-test). (g) Fine-tuning resistance to *P. syringae hopq^1-1^* in transgenic plants. Error bars represent the mean±SD; n=3; lowercase letters indicate statistical significance tested between multiple groups by one-way ANOVA at P<0.05. (h) Fine-tuning systemic acquired resistance to *P. syringae hopq^1-1^* in transgenic plants. Three lower leaves on each 4-week-old soil-grown plant were infiltrated with H_2_O or *P. syringae hopq^1-1^* suspension (OD=0.0001). Two systemic leaves were challenge-inoculated with a *P. syringae hopq^1-1^* suspension (OD=0.0001) at 24 hours after the last infiltration. Three days later, eight leaves were collected to examine pathogen growth. Error bars represent the mean±SD; n=3; lowercase letters indicate statistical significance tested between multiple groups by one-way ANOVA at P<0.05. (i) A model for the usage of uORFs_ACD11_ in precisely engineering crop disease resistance at required levels to minimize negative impacts on plant growth. uORFs_ACD11_ inhibits downstream gene translation in normal conditions and releases it upon pathogens. uORFs_ACD11_ coupled with *PR1* promoter can confer fine-tuned resistance in transgenic plants specifically when plants are facing pathogen threats.

Stable transgenic *N. benthamiana* lines expressing pPR1-NAT-LecRK-VI.2, pPR1-uorf1/2-LecRK-VI.2, or pPR1-uorf1/2/3-LecRK-VI.2 were created for functional analysis of the pPR1-uORFs_ACD11_ cassettes (Figure S5). Consistent with a previous report that transgenic *N. benthamiana* plants expressing AtLecRK-VI.2 exhibit normal growth phenotypes [42], no retarded growth was found in *N. benthamiana* lines expressing pPR1-NAT-LecRK-VI.2 or pPR1-uorf1/2-LecRK-VI.2 (Figures 7(a) and 7(b)). Notably, stable expression of pPR1-uorf1/2/3-LecRK-VI.2 in *N. benthamiana* resulted in growth inhibition (Figures 7(a) and 7(b)). Consistent with the case in *A. thaliana*, treatment of leaves with CF resulted in a significant induction of LecRK-VI.2 transcription levels in all three stable transgenic lines (Figure 7(c)). Upon inoculation of leaves using *P. capsici* mycelium (Figure 7(d)), LecRK-VI.2 protein accumulation also increased by variable levels in the three transgenic lines (Figure 7(e)). uorf1/2, NAT, and uorf1/2/3 showed relatively strong, moderate, and weak translational repression efficiencies, respectively (Figure 7(e)). These results indicate that the pPR1-uORFs_ACD11_ cassettes can fine-tune downstream gene expression and translation in a pathogen-inducible manner.

In resistance assay, all three stable transgenic lines exhibited enhanced resistance to both *P. capsici* (Figure 7(f)) and *Pst* DC3000 *hopq1-1^-^* (Figure 7(g)) as compared to wildtype *N. benthamiana* plants, with pPR1-uorf1/2/3-LecRK-VI.2 delivering strongest protection.

LecRK-VI.2 functions as an extracellular pyridine nucleotide receptor involved in SAR [41]. To test whether the observed alteration protein levels in the three transgenic line affected SAR response, we assessed the ability of DC3000 *hopq^1-1^* to colonize upper leaves three days after the inoculation of lower leaves. Systemic resistance was observed in wildtype and the three transgenic lines (Figure 7(h)). However, none of the three transgenic lines exhibited a proportionally stronger SAR response from controls (Figure 7(h)).

## 4. Discussion

Fine-tuning of quantitative traits is highly valued by breeders as it affords a sound approach to harness useful characteristics for breeding without serious field impairment [49, 50]. Naturally occurred weak alleles affecting important traits have contributed to great advances in domestication, evolution, and breeding [51, 52], but their utilization is restricted by low availability. Ectopic expression of foreign genes is an alternative approach to introduce the desired traits. However, overexpression can lead to undesirable phenotypes. For example, ectopic expression of AtNPR1 in rice using the maize ubiquitin promoter resulted in abnormal plant development and a reduction in seed size under some conditions [17]. Constitutive expression of the coding region of tobacco *tbz17* (*Nttbz17*) results in plants with thicker leaves [53]. Here, we successfully controlled the transgene products by using uORFs_ACD11_ and PR1 promoter to achieve a fine-tuned resistance level without growth retardation in transgenic plants (Figure 7(i)).

A uORF is a small ORF containing a start codon located upstream of their regulated gene. The three uORFs in 5${}^{\prime }$-UTR_ACD11_ are conserved among Arabidopsis accessions, indicating their important regulatory roles. Their variable translational repression efficiencies may at least partially depend on the Kozak sequence context around their start codons [54]. Despite their relatively low Kozak strength, the three uORFs_ACD11_ effectively repress the translation of downstream gene in a redundant manner. Among them, uORF3 has the shortest length of 9 base pairs but the most favorable Kozak strength, which leads to its strongest translational repression efficiency. This observation explains why the uorf1/2 cassette has strong repression capacity and is suitable for fine-tuning transgene expression and balancing plant growth and immunity.

Natural uORFs has been successfully used in engineering plant defense. Ectopic expression of AtNPR1 driven by 35S:uORFs_AtTBF1_ renders broad-spectrum disease resistance to rice plants with no apparent growth retardation [34]. The potential value of edited uORFs is also emerging gradually. For example, uORF engineering of an important enzyme in vitamin C synthesis, LsGGP2, improves the tolerance of lettuce to oxidation stress and ascorbate content [55]. Here, we have shown that a modified uORF, uORFs_ACD11_, can be used to down regulate gene translation under normal growth conditions while enabling the activation of translation when a pathogen is detected.

*ACD11* encodes a phingosine transfer protein. Its knockout causes activation of defense response and programmed cell death (PCD), indicating that ACD11 negatively regulates plant immunity [35]. The protein stability of ACD11 is also regulated by the E3 ligase XBA35.2 and its binding partners of BPL family proteins [36, 37]. The three uORFs characterized in this study may be an additional control layer of ACD11 protein accumulation. In plants, the protein levels of such negative regulators are strictly controlled by multiple layers of mechanisms. Other examples of this multilayered regulation include the pentatricopeptide repeats protein-like (PPRL) protein negatively regulates RESISTANT TO *P. SYRINGAE* 2- (RPS2-) mediated resistance pathway and is downregulated by a natural antisense short interfering RNA (nat-siRNA) derived from *Arabidopsis GTP-BINDING 2* (*ATGB2*) gene (nat-siRNAATGB2) at the transcription level [56]. EDS1-interacting J protein 1 (EIJ1) plays a negative role in plant defense response and its protein accumulation is restricted during pathogen infection process [57].

Based on our data, uORFs_ACD11_-mediated translational repression was attenuated upon either PAMP treatment or pathogen inoculation. Similar phenomenon has been reported in multiple other genes such as uORF_TBF1_ [31, 58, 59]. The R-motif, a highly enriched consensus sequence consisting of mostly purines, can be found in the 5${}^{\prime }$-UTR of these genes. However, no R-motif can be identified from 5${}^{\prime }$UTR_ACD11_, indicating that *ACD11* and R-motif containing genes like *TBF1* may respond to pathogen infection via distinct mechanisms. Additionally, the translational repression ability of uORFs_ACD11_ become weaker after the avirulent strain treatment. Avirulent strains lead to effector-triggered immunity (ETI) in host cells. Compared with basal resistance, ETI induces a stronger and faster defense response against pathogens. We speculated that the inhibition of uORFs_ACD11_-mediated translational repression ability was a part of defense response, and thus, uORFs_ACD11_-mediated translational repression was weaker upon avirulent strains.

The integration of *pPR1* is an improvement to the uORFs_ACD11_ cassettes. The adoption of pathogen-inducible promoters is a logical solution to reduce growth distortion triggered by defense-related transgenes [19, 20], but they should be carefully selected for genetic engineering to avoid those with unfavorable auto-activation effect [24, 25]. In this study, no obvious auto-activation is detected for *pPR1*-uORFs_ACD11_ cassettes and the strategy successfully avoids potential growth retardation induced by AtLecRK-VI.2.

In this study, we fine-tune AtLecRK-VI.2 expression via the combinations of pPR1 and uORFs_ACD11_ cassettes. In this way, AtLecRK-VI.2 expression is strictly induced by pathogen infection and optimized to provide satisfactory resistance protection with no apparent impair on plant growth. These cassettes can be used for fine-tuning other genes of interest. They would be a useful toolkit for precisely engineering crop disease resistance and other important agronomic traits.

## Data Availability

The TAIR locus IDs for genes mentioned in this study are AT5G01540 (AtLecRK-VI.2), AT2G34690 (AtACD11), and AT4G36990 (AtTBF1).

## Additional Points

*Highlight*. The uORFsACD11 cassettes are demonstrated to be a useful toolkit for engineering crop disease resistance to desired levels and minimizing negative impact towards plant growth.

## Supplementary Materials

Supplementary Materials Figure S1: no interference is detected between green fluorescence and luminescence. Leaves were agro-infiltrated with GFP or LUCIFERASE for 48 hours. Photos were taken by a cooled charge-coupled imaging apparatus. Figure S2: the three uORFs identified in the 5${}^{\prime }$-UTR of ACD11. (a) Detail sequence information of the three uORFs. (b) Kozak strength of uORFsACD11 and mORFACD11. Different start codon types with related Kozak strength are shown in the left. Start codon sequences with related Kozak strength is shown in the right. Figure S3: variation of uORFsACD11 in 1,135 Arabidopsis accessions. (a) Percentages of different uORF types in 1,135 Arabidopsis accessions. (b) uORF2 sequences of Col-0 and 10 popular accessions. Figure S4: validation of transgenic Arabidopsis. (a) Validation of transgenic Arabidopsis. The designation of primers is shown at the top. PCR results using the primers is shown below. (b) GFP signal of transgenic leaves detected by in vivo fluorescence imaging system. Bright field image is shown on the left. Image taken by GFP channel is shown on the right. (c) A schematic diagram illustrating the experiment designation. Figure S5: validation of transgenic plants. The designation of primers is shown at the top. PCR results using the primers are shown below. Table S1: list of primers.
