# Supplementary material for: Making Use of Plant uORFs to Control Transgene Translation in Response to Pathogen Attack
Source: Biodes Res. 2022 Feb 3;2022:9820540. doi: 10.34133/2022/9820540 (PMC10521741; doi:10.34133/2022/9820540)
Supplement: Supplementary Materials — Figure S1: no interference is detected between green fluorescence and luminescence. Leaves were agro-infiltrated with GFP or LUCIFERASE for 48 hours. Photos were taken by a cooled charge-coupled imaging apparatus. Figure S2: the three uORFs identified in the 5′-UTR of ACD11. (a) Detail sequence information of the three uORFs. (b) Kozak strength of uORFsACD11 and mORFACD11. Different start codon types with related Kozak strength are shown in the left. Start codon sequences with related Kozak strength is shown in the right. Figure S3: variation of uORFsACD11 in 1,135 Arabidopsis accessions. (a) Percentages of different uORF types in 1,135 Arabidopsis accessions. (b) uORF2 sequences of Col-0 and 10 popular accessions. Figure S4: validation of transgenic Arabidopsis. (a) Validation of transgenic Arabidopsis. The designation of primers is shown at the top. PCR results using the primers is shown below. (b) GFP signal of transgenic leaves detected by in vivo fluorescence imaging system. Bright field image is shown on the left. Image taken by GFP channel is shown on the right. (c) A schematic diagram illustrating the experiment designation. Figure S5: validation of transgenic plants. The designation of primers is shown at the top. PCR results using the primers are shown below. Table S1: list of primers. [file 9820540.f1.zip › uORF_FigureS1-52022.1.9.pptx]

## Slide 1
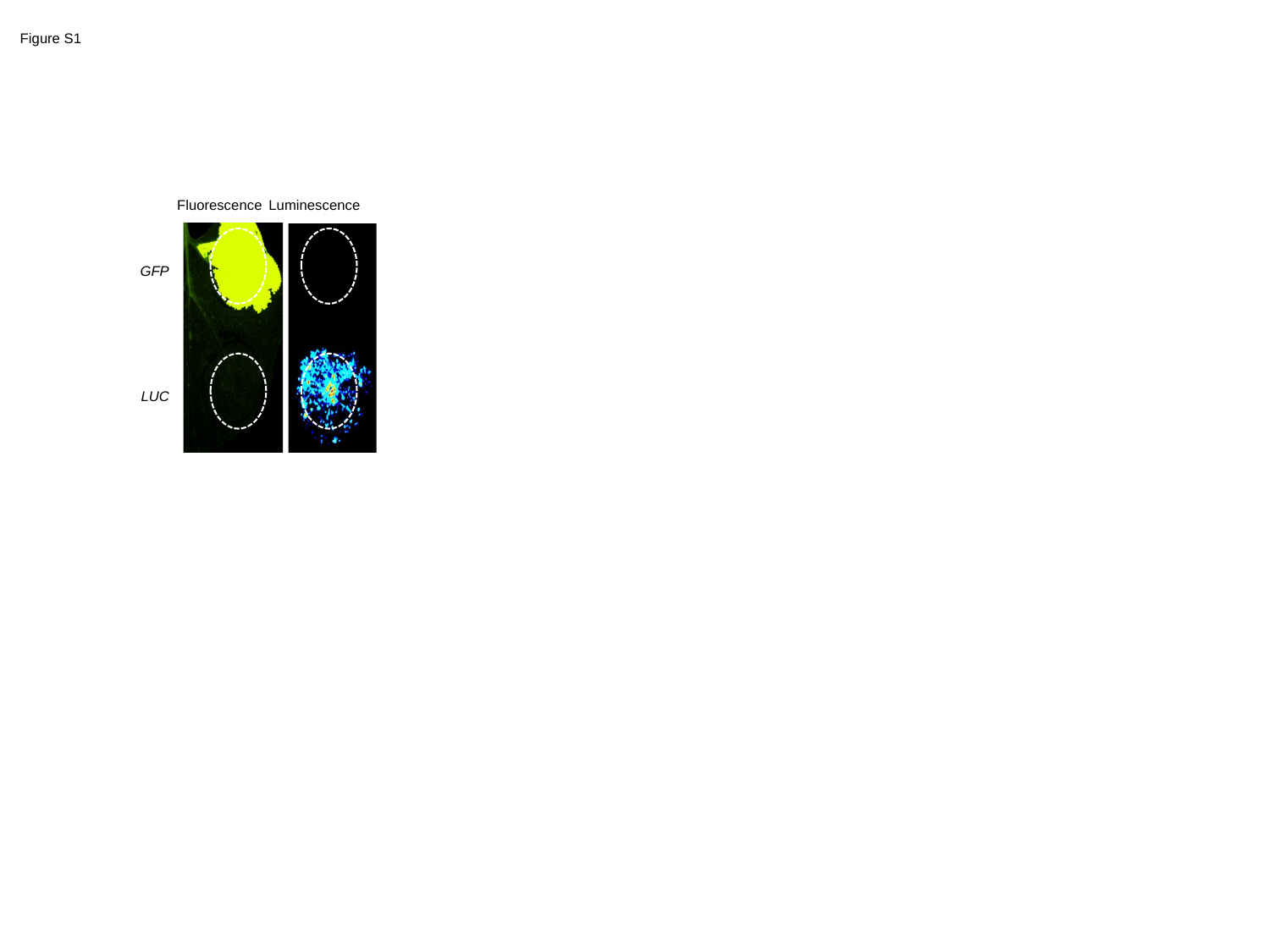

Figure S1
Fluorescence
Luminescence
GFP
LUC

## Slide 2
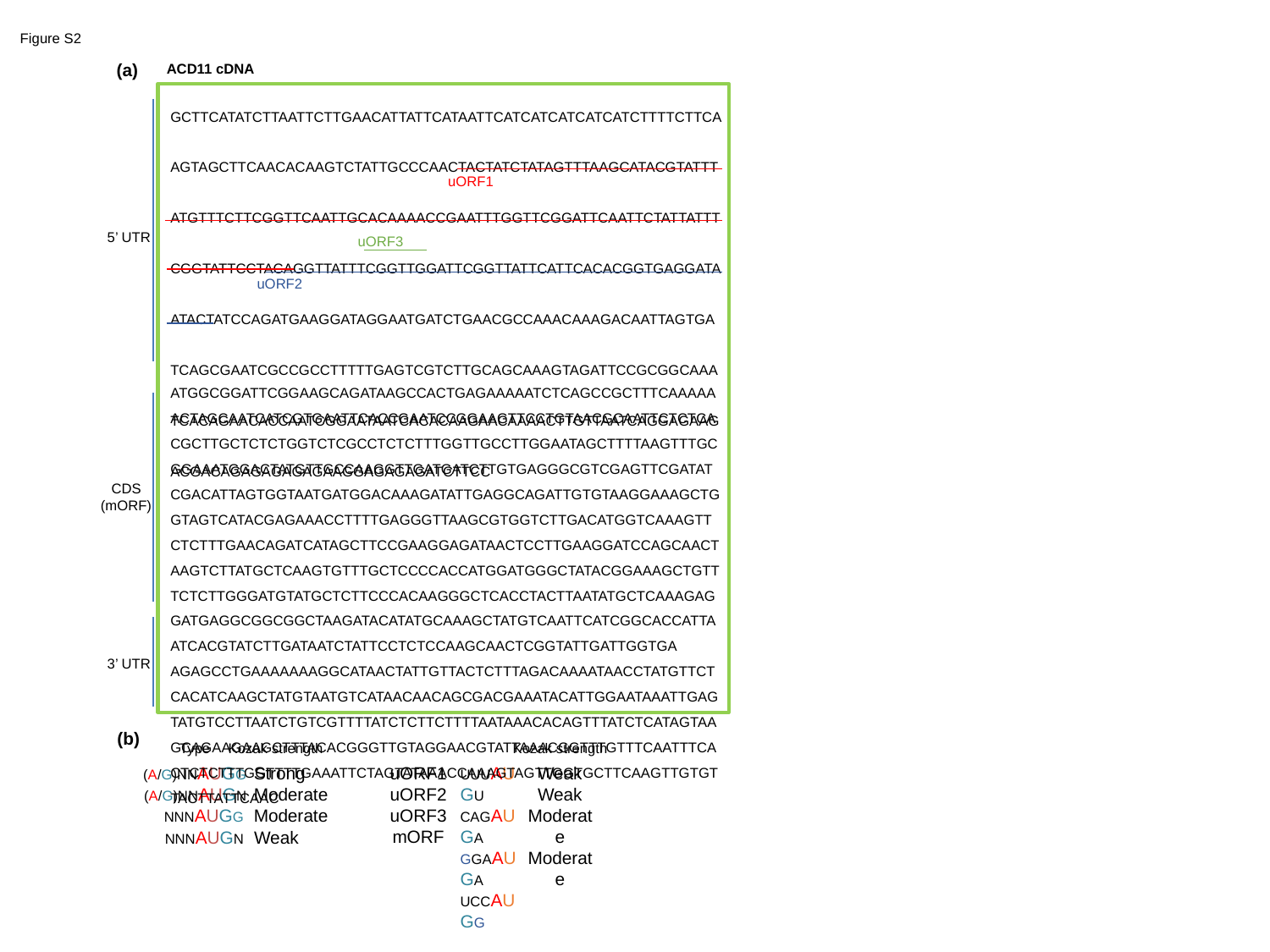

Figure S2
(a)
ACD11 cDNA
GCTTCATATCTTAATTCTTGAACATTATTCATAATTCATCATCATCATCATCTTTTCTTCAAGTAGCTTCAACACAAGTCTATTGCCCAACTACTATCTATAGTTTAAGCATACGTATTTATGTTTCTTCGGTTCAATTGCACAAAACCGAATTTGGTTCGGATTCAATTCTATTATTTCGGTATTCCTACAGGTTATTTCGGTTGGATTCGGTTATTCATTCACACGGTGAGGATAATACTATCCAGATGAAGGATAGGAATGATCTGAACGCCAAACAAAGACAATTAGTGATCAGCGAATCGCCGCCTTTTTGAGTCGTCTTGCAGCAAAGTAGATTCCGCGGCAAATCACAGAACACCAATCGGAATAATCACACAAGAACAAAACTTGTTAATCAGGAGAAGACGACAGAGAGAGAGAAGGAGAGAGATCTTCC
uORF1
5’ UTR
uORF3
uORF2
ATGGCGGATTCGGAAGCAGATAAGCCACTGAGAAAAATCTCAGCCGCTTTCAAAAAACTAGCAATCATCGTGAATTCACCGAATCCGGAAGTTCCTGTAACGCAATTCTCTCACGCTTGCTCTCTGGTCTCGCCTCTCTTTGGTTGCCTTGGAATAGCTTTTAAGTTTGCGGAAATGGACTATGTTGCCAAGGTTGATGATCTTGTGAGGGCGTCGAGTTCGATATCGACATTAGTGGTAATGATGGACAAAGATATTGAGGCAGATTGTGTAAGGAAAGCTGGTAGTCATACGAGAAACCTTTTGAGGGTTAAGCGTGGTCTTGACATGGTCAAAGTTCTCTTTGAACAGATCATAGCTTCCGAAGGAGATAACTCCTTGAAGGATCCAGCAACTAAGTCTTATGCTCAAGTGTTTGCTCCCCACCATGGATGGGCTATACGGAAAGCTGTTTCTCTTGGGATGTATGCTCTTCCCACAAGGGCTCACCTACTTAATATGCTCAAAGAGGATGAGGCGGCGGCTAAGATACATATGCAAAGCTATGTCAATTCATCGGCACCATTAATCACGTATCTTGATAATCTATTCCTCTCCAAGCAACTCGGTATTGATTGGTGA AGAGCCTGAAAAAAAGGCATAACTATTGTTACTCTTTAGACAAAATAACCTATGTTCTCACATCAAGCTATGTAATGTCATAACAACAGCGACGAAATACATTGGAATAAATTGAGTATGTCCTTAATCTGTCGTTTTATCTCTTCTTTTAATAAACACAGTTTATCTCATAGTAAGCAGAAGAAGCTTTACACGGGTTGTAGGAACGTATTAAACGGTTTGTTTCAATTTCACTCTCTTTGGTTTTGAAATTCTAGTATAAACCAAAGTAGTTGGTGCTTCAAGTTGTGTTACTTATTCAAC
CDS
(mORF)
3’ UTR
(b)
Kozak strength
Kozak strength
Type
(A/G)NNAUGG
Strong
uORF1
uORF2
uORF3
mORF
UUUAUGU
CAGAUGA
GGAAUGA
UCCAUGG
Weak
Weak Moderate
Moderate
(A/G)NNAUGN
Moderate
NNNAUGG
Moderate
NNNAUGN
Weak

## Slide 3
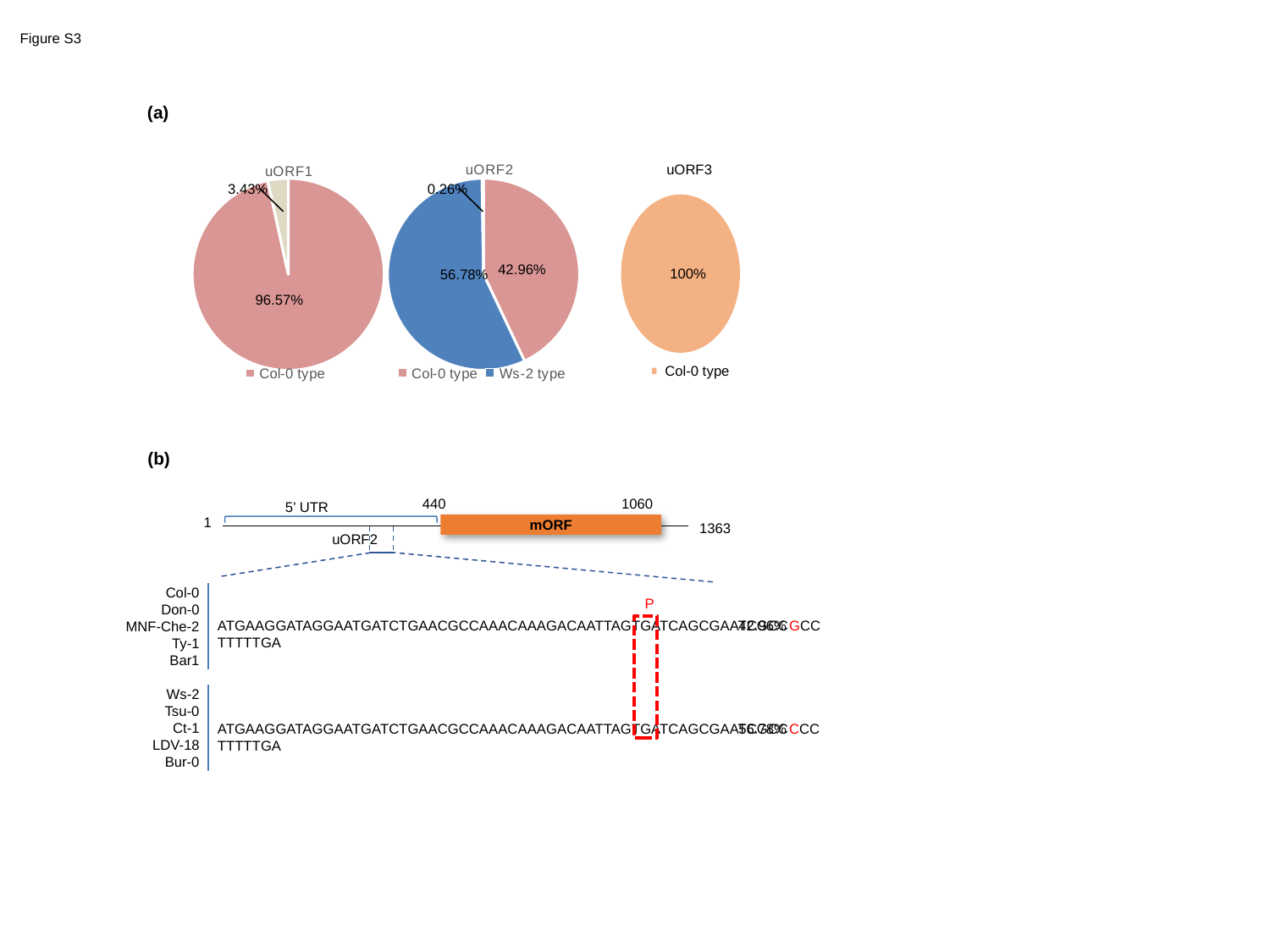

Figure S3
(a)
### Chart: uORF2
| Category | 销售额 |
|---|---|
| Col-0 type | 42.96 |
| Ws-2 type | 56.78 |
| Other type | 0.26 |
### Chart: uORF1
| Category | 销售额 |
|---|---|
| Col-0 type | 96.57 |
| Other type | 3.43 |uORF3
3.43%
0.26%
42.96%
100%
56.78%
96.57%
Col-0 type
(b)
440
1060
1
1363
mORF
uORF2
Col-0
Don-0
MNF-Che-2
Ty-1
Bar1
Ws-2
Tsu-0
Ct-1
LDV-18
Bur-0
5’ UTR
P
ATGAAGGATAGGAATGATCTGAACGCCAAACAAAGACAATTAGTGATCAGCGAATCGCCGCCTTTTTGA
42.96%
ATGAAGGATAGGAATGATCTGAACGCCAAACAAAGACAATTAGTGATCAGCGAATCGCCCCCTTTTTGA
56.78%

## Slide 4
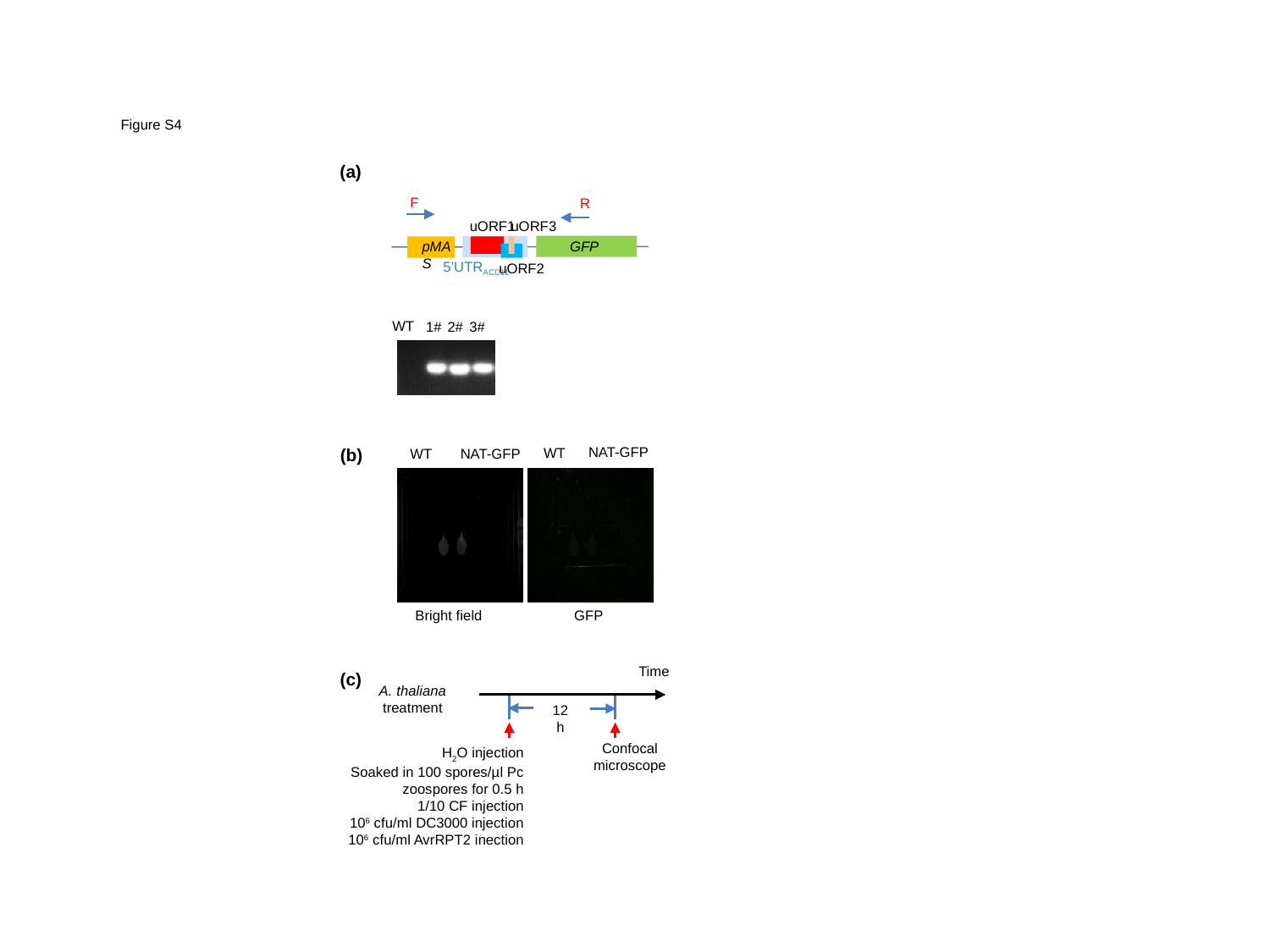

Figure S4
(a)
F
R
uORF1
uORF3
pMAS
GFP
5’UTRACD11
uORF2
WT
1#
2#
3#
NAT-GFP
(b)
WT
NAT-GFP
WT
Bright field
GFP
Time
(c)
A. thaliana
treatment
12 h
Confocal microscope
H2O injection
Soaked in 100 spores/µl Pc zoospores for 0.5 h
1/10 CF injection
106 cfu/ml DC3000 injection
106 cfu/ml AvrRPT2 inection

## Slide 5
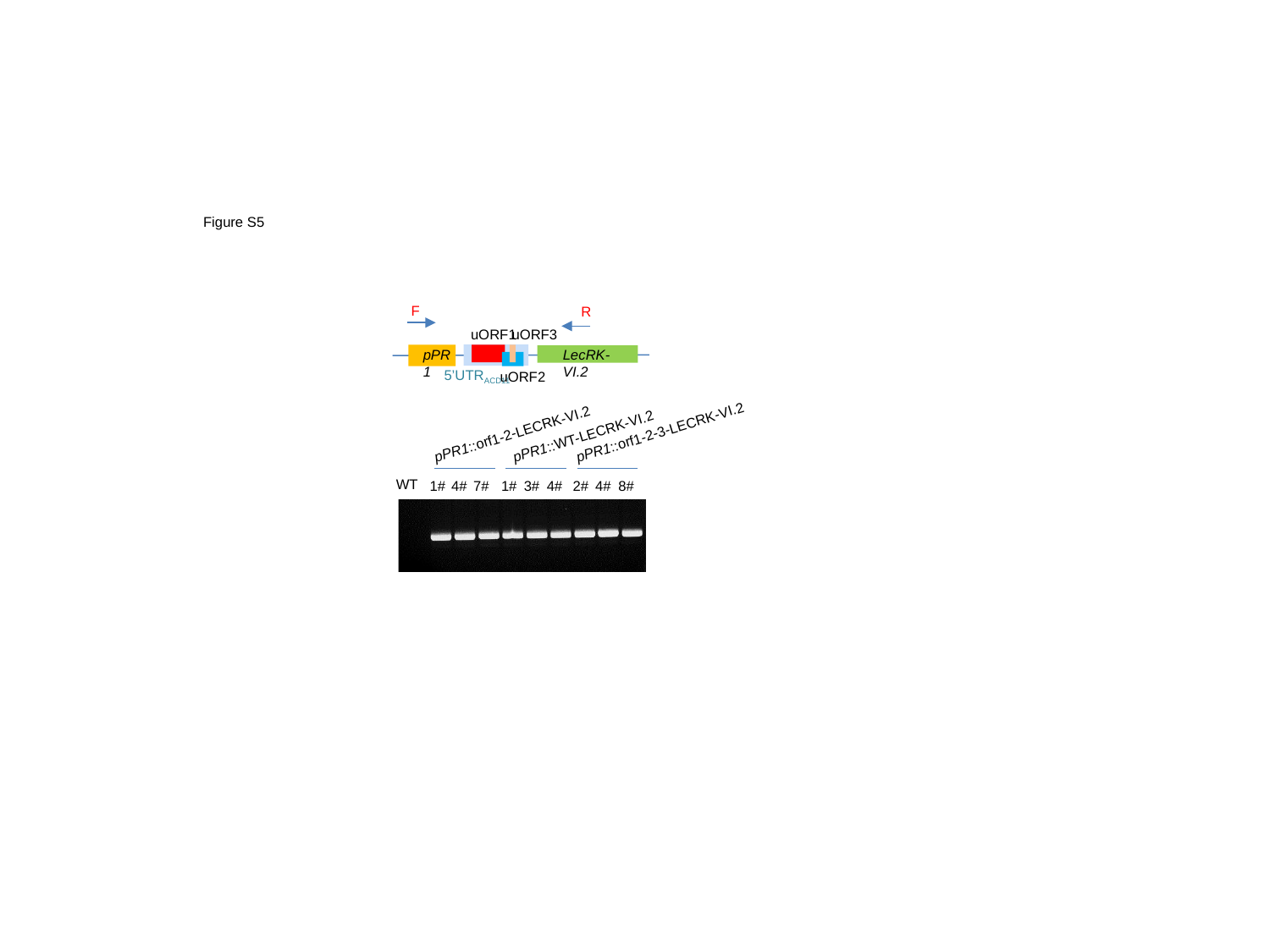

Figure S5
F
R
uORF1
uORF3
pPR1
LecRK-VI.2
5’UTRACD11
uORF2
pPR1::orf1-2-3-LECRK-VI.2
pPR1::orf1-2-LECRK-VI.2
pPR1::WT-LECRK-VI.2
WT
1#
4#
7#
1#
3#
4#
2#
4#
8#
